# Mitochondrial respiratory dysfunction due to the conversion of substituted cathinones to methylbenzamides in SH-SY5Y cells

**DOI:** 10.1038/srep14924

**Published:** 2015-10-14

**Authors:** Bjørnar den Hollander, Mira Sundström, Anna Pelander, Antti Siltanen, Ilkka Ojanperä, Eero Mervaala, Esa R. Korpi, Esko Kankuri

**Affiliations:** 1 Department of Pharmacology, Faculty of Medicine, Biomedicum Helsinki, Haartmaninkatu 8, FI-00014 University of Helsinki, Finland; 2 Department of Forensic Medicine, Kytösuontie 11, FI-00014 University of Helsinki, Finland

## Abstract

The increased use of cathinone-type designer drugs, known as legal highs, has led to concerns about their potential neurotoxicity due to their similarity to methamphetamine (METH). Therefore, closer investigations of their toxic effects are needed. We investigated the effects of the cathinones 4-methylmethcathinone (4-MMC) and 3,4-methylenedioxymethcathinone (MDMC) and the amphetamine METH on cytotoxicity and mitochondrial respiration in SH-SY5Y neuroblastoma cells. We also investigated the contribution of reactive species, dopamine, Bcl-2 and tumor necrosis factor α (TNFα) on toxicity. Finally, we investigated the effect of cathinone breakdown products using ultra-high performance liquid chromatography/high-resolution time-of-flight mass spectrometry and studied their involvement in toxicity. We observed dose-dependent increases in cytotoxicity and decreases in mitochondrial respiration following treatment with all cathinones and amphetamines. Glutathione depletion increases amphetamine, but not cathinone toxicity. Bcl-2 and TNFα pathways are involved in toxicity but dopamine levels are not. We also show that cathinones, but not amphetamines, spontaneously produce reactive species and cytotoxic methylbenzamide breakdown products when in aqueous solution. These results provide an important first insight into the mechanisms of cathinone cytotoxicity and pave the way for further studies on cathinone toxicity *in vivo*.

The term “legal highs” is commonly used to refer to a range of novel psychoactive substances that are sold for recreational use. However, little is known yet about their potential neurotoxicities and long-term effects. A class of substances known as substituted cathinones is of particular interest. Substituted cathinones include substances such as 4-methylmethcathinone (4-MMC, “mephedrone”) and 3,4-methylenedioxymethcathinone (MDMC, “methylone”)^1^,^2^. These cathinones are closely related, in their chemical structure and in their pharmacological actions, to those of the amphetamines methamphetamine (METH) and 3,4-methylenedioxymethamphetamine (MDMA, “ecstasy”)^3^,^4^. It is known that amphetamines can produce neurotoxicity by several mechanisms, such as increasing levels of oxidative stress and inhibiting mitochondrial function^5^. This raises an essential question as to whether the substituted cathinones work by similar mechanisms to produce effects involved in neurotoxicity.

Recent studies show that cathinones are less likely than amphetamines to produce persistent decreases in markers of neurotoxicity^3^,^6^. Such markers include brain dopamine (DA), DA transporters (DAT) or the DA biosynthesis enzyme tyrosine hydroxylase (TH) in rodents^3^,^6^. On the other hand, cathinones appear to potentiate the toxicity of amphetamines when the two are co-administered. Additionally, they may be toxic when administered at high ambient temperatures, and effects of cathinones on memory function have also been observed^7^,^8^,^9^. Furthermore, recent studies show that cathinones can produce similar toxicity as amphetamines *in vitro*. For instance, Araujo *et al.*^10^ reported cytotoxicity in response to both amphetamines and cathinones in primary cultured rat hepatocytes. Moreover, we showed previously that cytotoxicity and decreases in mitochondrial respiration occur in response to both cathinones and amphetamines in cultured neuronal cells^11^. Cytotoxicity following both cathinones and amphetamines administration was also observed in Chinese hamster ovary cells that had been transfected with monoamine transporters^12^.

In order to understand better the potential risks for human cathinone users and the discrepancies between *in vivo* and *in vitro* derived data, more detailed investigations into the mechanisms involved in cytotoxic effects of cathinones are required. A few years ago, the interesting observation was made that the cathinone 4-MMC molecule rapidly breaks down into *N*-4-dimethylbenzamide (DMBA) under alkaline conditions^13^. Subsequently, we demonstrated that MDMC also breaks down into its corresponding 3,4-methylenedioxy-N-methylbenzamide (MDMBA) and that this breakdown can also effectively occur at physiological pH (pH 7.35) in the presence of an electron acceptor^11^. However, the contribution of these methylbenzamides to neuronal cell toxicity remains to be determined.

Here, we explore and compare mechanisms involved in the cytotoxic response to cathinones and their methylbenzamide breakdown products with those of typical amphetamines. For this purpose, we used the SH-SY5Y neuroblastoma cell line as this cell line shows many neuronal characteristics and is a well-established model in research on amphetamine neurotoxicity^14^,^15^. Our broad objective was to study toxicity for DA producing and non-DA producing cells, thus we describe experiments using an undifferentiated cell line and also cells that had been subjected to a differentiation protocol known to induce a specific DA neuron-like phenotype^16^. Our principal focus was on the effects of cathinones and amphetamines on cytotoxicity and mitochondrial function in addition to studying the mechanisms that include reactive oxygen species (ROS) and tumor necrosis factor α (TNF α). Importantly, we demonstrate that the methylbenzamide breakdown products that are produced from cathinones in aqueous solutions under normal physiological conditions may contribute to the observed effects of cytotoxicity and mitochondrial dysfunction.

## Methods

### Drugs and reagents

Racemic 4-MMC and MDMC were acquired from the Department of Forensic Medicine, Hjelt Institute, University of Helsinki. The compounds were analyzed for purity by gas chromatography and Fourier transform infrared spectroscopy and found to be pure (>95%) hydrochloride salts. MDMA (catalog ID THC-1347) was acquired from THC Pharm GmbH (Frankfurt, Germany); DMBA (catalog ID H56446) from Alfa Aesar GmbH (Karlsruhe, Germany) and MDMBA (catalog ID 10W-0825 from Key Organics (Camelford, UK). METH (catalog ID M8750) and other reagents, unless otherwise specified, were acquired from Sigma-Aldrich (St. Louis, MO, USA) and were all of analytical quality. All drugs used in the experiments were dissolved in their respective vehicle (growth medium, water or phosphate-buffered saline) solutions. The DMBA and MDMBA drugs are not water-soluble thus they were dissolved in a vehicle containing 1% dimethyl sulfoxide.

### SH-SY5Y cell culture

SH-SY5Y neuroblastoma cells were obtained from ATCC (Middlesex, UK). They were maintained between passages 6 and 20 in Dulbecco’s Modified Eagle Medium (DMEM/F12), which contains 10% fetal bovine serum, penicillin (100 U/ml), streptomycin (100 μg/ml) and amphotericin B (0.25 μg/ml) (all obtained from Gibco - Invitrogen, Paisley, UK) and kept in an incubator at a temperature of 37 °C with 5% CO_2_. Experiments were done on undifferentiated cells and also on cells differentiated by a 3-day treatment with 10 μM retinoic acid followed by a 3-day treatment with 80 nM 12-*O*-tetradecanoylphorbol-13-acetate (RA-TPA-differentiated cells), which is known to induce a more dopamine neuron-like phenotype^16^.

### Cytotoxicity assays

SH-SY5Y cells were plated on 96-well plates at a density of ±5000 cells/100 μL well and grown for 4 or 6 days (RA-TPA-differentiated cells) before the medium was replaced with medium containing one of the following: METH, MDMA, 4-MMC or MDMC (2.5–2000 μM). Cytotoxicity of each drug molecule was determined by assessing the amount of lactate dehydrogenase (LDH) released into the growth medium, which was then assayed by the Cytotoxicity Detection Kit (Roche, Basel, Switzerland) according to the manufacturer’s instructions. The LDH concentrations in the dose-response experiments and buthionine sulfoximine (BSO) experiments were measured in the cells and also the medium at 24 h and 48 h after the drug treatment. The LDH release was calculated by dividing the LDH content in the medium by the total amount of LDH in medium and the cells at both time points. The mean values of the two measurements are shown. Other determinations of LDH for each drug treatment were made at 48 h only. In experiments where drug treatments were combined with the DAT blocker GBR12909 (1 μM), the cells were pre-treated for 1 h with GBR12909 prior to application of the drug in combination with GBR12909. In experiments where drug treatments were combined with BSO, the cells were treated with BSO for 24 h prior to application of the drug in combination with BSO.

### Bcl-2 overexpression

SH-SY5Y cells were plated at 10,000/cm^2^ for transfection. The cells were transfected 24 h later with the retroviral vector that carries the human BCL-2 gene (pBABEpuro, Biomedicum Functional Genomics Unit, University of Helsinki). Transfection was carried out with 8 μg/ml polybrene at 1200 g centrifugation for 1 h. Incubation was continued at 37 °C for 6 h, after which 2 volumes of culture medium was added to reduce the polybrene toxicity The procedure was repeated 24 h later to increase transfection efficiency. The cells were incubated with 1 μg/ml puromycin for 48 h to increase the ratio of BCL-2-transfected cells. Puromycin selection was repeated during the next passage. The antiapoptotic function of this construct has been previously validated and the overexpression of the Bcl-2 does not influence the expression of other genes^17^.

### Analysis of gene expression

Total RNA was isolated from undifferentiated and RA-TPA differentiated SH-SY5Y cells by using the RNEasy Mini Kit (Qiagen, Hilden, Germany). Sample RNA integrity and quality were confirmed by the Agilent Bioanalyzer 2100 (Agilent Technologies, Santa Clara, CA, USA). 100 ng of total RNA was primed using oligo-dT(T7) primers and converted to cDNA with SuperScript III Reverse Transcriptase. cDNA was then converted to double-stranded cDNA using DNA Polymerase (Life Technologies, Carlsbad, CA, USA). The double-stranded cDNA template was amplified and labeled by *in vitro* transcription to generate multiple copies of biotinylated cRNA. Biotinylated cRNA was purified by RNeasy Mini Kit and was hybridized to HumanHT-12 v4 Expression BeadChips (Illumina Inc. San Diego, CA, USA) for 18 hours at 58 °C. BeadChips were washed, blocked and stained with streptavidin-Cy3 and scanned by using the Illumina iScan (Illumina Inc.), according to the manufacturer’s protocol. The raw data were extracted using Illumina GenomeStudio software. The gene expression fold-changes were calculated from the background-corrected and the quantile-normalized data.

### TNFα measurement

The TNFα levels of the cells were measured by using the AlphaLISA Human TNFα Research Immunoassay Kit by PerkinElmer (Waltham, MA). Cells were grown normally and treated with various drugs for 48 h as described above. Subsequently, TNFα content in the medium was measured according to the manufacturer’s instructions. Briefly, 5 μL of medium was placed in wells of a 96-well AlphaLISA assay plate and subsequently, acceptor beads (final concentration: 10 μg/ml) were added to the wells and the plate was incubated for 30 min at 23 °C. Next, anti-analyte antibody (final concentration: 1 nM) was added and the plate incubated for 60 min. Finally, donor beads (final concentration: 40 μg/ml) were added to the wells and the plate incubated for another 30 min before the final readout was made on an Envision Multilabel Reader (PerkinElmer). Concentrations of TNFα in the medium were calculated when their plots were read against those of standard curves produced by using the manufacturer-provided TNFα.

### Western Blotting

Western blotting was carried out as detailed previously^18^,^19^. Briefly, cells were cultured on 96-well plates as detailed above. Cells with or without retinoic acid differentiation received either 2 mM mephedrone or vehicle. After 48 hours, cells in each well were harvested into 75 μl cell lysis buffer (1% Triton-X100, 20 mM Tris Base, 100 mM NaCl, 1 mM EDTA, pH adjusted to 7.4; one tablet of complete Mini EDTA-free Protease inhibitors (Roche Diagnostics GmbH, Mannheim, Germany was added to 10 ml of buffer). Cells from 8 wells were pooled as one sample. The samples were sonicated for 3 × 10 s, and protein content was analyzed using the Pierce™ BCA Protein Assay Kit (Thermo Scientific, Rockford, IL). Protein content in samples from each experiment was equalized by diluting the samples with high protein content to the protein concentration of the most dilute sample in appropriate amounts of lysis buffer and 5x concentrated sample buffer (Lane Marker Reducing Sample Buffer, cat#3900, Thermo Scientific). Samples were heated in a block-heater at 97 °C for 5 min, and were stored at −20 °C. For western blotting samples were thawed and re-heated at 97 °C for 2 min. Controls and samples from a single experiment were run on the same gel to enable comparative analysis. An equal sample volume of 20 μl was added to each well of Mini-Protean® TGX™ 4–20% Precast Gels (Bio-Rad, Hercules, CA). Electrophoresis was carried out in Tris/Glycine/SDS buffer (Bio-Rad #161-0732) followed by blotting to nitrocellulose membranes in transfer buffer (containing 25 mM Tris, 192 mM glycine, 20% methanol, pH 8.3) as detailed previously^18^,^19^ After blotting membranes were incubated for 1 h in 2.5% skimmed milk powder Tris buffer pH7.5 followed by a 2 h-incubation with the primary antibody (p53: mouse anti-p53: clone DO-1, sc-126 and rabbit anti-p53, sc-6243 were used for verification of results in consecutive runs; both from Santa Cruz Biotech, Dallas, TX). Membranes were then incubated for 1 h with secondary antibodies (goat-anti-rabbit IRDye® 800CW and goat-anti-mouse IRDye® 680LT; both from Li-Cor Biosciences, Lincoln, NE). Signal detection was carried out using Li-Cor Odyssey system and ImageStudio 2.0.38 software (both from Li-Cor Biosciences, Lincoln, NE). Quantification of band intensity in relative fluorescence units was carried out using the gel analysis feature of Fiji software^20^. Fold-changes between band intensities of controls and samples on same membranes were compared assigning the control band intensity to 1.

### Fluorometric detection of H_2_O_2_ in Oxygraph-2k

The H_2_O_2_ production levels that resulted from METH, MDMA, 4-MMC or MDMC in aqueous solution were determined by real-time fluorometric detection of H_2_O_2_ using an Amplex Red/horseradish peroxidase (HRP) assay in an Oxygraph-2k respirometer instrument that was fitted with a fluorescence module (Oroboros Instruments GmbH, Innsbruck, Austria). Prior to the start of the assay, Amplex Red and HRP (final concentrations of 5 μM and 10 U/ml, respectively) were added to the chambers of the Oxygraph-2k instrument. After obtaining a steady 5 min baseline, known quantities of one of the following: METH, MDMA, 4-MMC, MDMC (final concentrations of 20 mM) or water were added to the Oxygraph-2k chambers and the changes in fluorescence over the next 5 min were measured. Drug-induced increases in cumulative H_2_O_2_ production were determined by dividing the slope measured after drug injections by the slope obtained prior to the injection of drugs into the chambers.

### Assessment of mitochondrial function

The effects of the drugs on the mitochondrial oxygen consumption rate (OCR) in the SH-SY5Y cells were assessed using a Seahorse Bioscience XF^e^96 analyzer (Massachusetts, USA) in combination with the Seahorse Bioscience XF Cell Mito Stress Test assay kit. In this assay, subsequent additions of the ATP synthase inhibitor oligomycin, the mitochondrial uncoupler carbonyl cyanide 4-(trifluoromethoxy)phenylhydrazone (FCCP) and the complex I + II inhibitors rotenone + antimycin A into the assay medium provided insight into different aspects of mitochondrial function. Experiments were performed on intact, adherent cells and also on permeabilized cells.

#### Intact cells

Cells were plated at a density of ±10,000 cells/80 μL well in 96-well assay plates (Seahorse Bioscience) into standard growth medium for SH-SY5Y cells and grown for 4 days or allowed to differentiate into RA-TPA-differentiated cells for 6 days before the medium was replaced by a medium containing one of the following: METH, MDMA, 4-MMC or MDMC (0.5–2 mM). After 24 h, the normal medium was replaced by assay medium consisting of XF Base Medium (Seahorse Bioscience) with added 10 mM glucose, 10 mM pyruvic acid, and 1 mM L-glutamine. Subsequently, the analysis of OCR was performed in a Seahorse XF^e^96 analyzer according to the manufacturer’s instructions. The OCR values were obtained during baseline (prior to addition of any Mito Stress Test substances) and after the addition of 1 μM oligomycin, 0.5 mM FCCP and 1 μM rotenone +1 μM antimycin A. Prior to analysis, data were corrected by withdrawing non-mitochondrial respiration (measured after the injection of rotenone + antimycin A) from all measured OCR values.

#### Permeabilized cells

Undifferentiated cells were plated at a density of ±40,000 cells/80 μL well in 96-well assay plates (Seahorse Bioscience) into a normal growth medium and grown for 24 h before the growth medium was replaced by a mannitol-sucrose buffer that consisted of 220 mM mannitol, 70 mM sucrose, 10 mM KH_2_PO_4_, 5 mM MgCl_2_, 2 mM HEPES and 1 mM EGTA in addition to 10 mM glutamate, 2 mM malate, 5 mM pyruvate, 1 mM ATP, 4 mM ADP. Furthermore, 4 mg/ml bovine serum albumin and 2 nM recombinant perfringolysin O (XF Plasma Membrane Permeabilizer, Seahorse Bioscience) were added to the buffer directly prior the start of the experiments. After obtaining baseline measurements were taken the drugs (METH, 4-MMC, DMBA, MDMC and MDMBA, all 2 mM) were added to the wells according to the study allocation and allowed to incubate for 10 min prior to the addition of 5 μM oligomycin, 5 μM FCCP and 2 μM rotenone +2 μM antimycin A.

### Analysis of DA and cathinone breakdown products

#### Dopamine

DA levels were assessed using high-performance liquid chromatography with electrochemical detection (HPLC-ED) under conditions similar to those described previously^8^. Cells were plated into 6-well plates and grown or differentiated normally. Additionally, RA-TPA-differentiated cells were also grown in the medium with 0.5 mM α-methyl-para-tyrosine added during the last 3 days of differentiated together with the TPA. Following the growth and differentiation protocols, cells from 3 wells were pooled into one sample which was prepared by brief sonication in 2% perchloric acid and centrifugation (at 15,000 g for 30 min). Subsequently, 10 μL of supernatant was injected into the HPLC system and analyzed for the DA content. The mobile phase consisted of purified water with 8% methanol, 50 mM citric acid, 1.5 mM 1-octanesulfonic acid, 0.05 mM EDTA and 50 mM phosphoric acid. The retention time for DA was 7.45 min, and DA concentrations were calculated by being read against linear standard curves.

#### Cathinone breakdown product

The formation of the MDMC breakdown product MDMBA was assessed by dissolving the compounds in a pH 12 buffer and measuring the level of breakdown product following 0, 24, and 48 h incubation at 37 °C. The amount of 4-MMC breakdown product DMBA in cellular growth medium following a 48 h treatment of SH-SY5Y cells with 4-MMC both with and without GBR12909 was also measured. An ultra-high performance liquid chromatography/high-resolution time-of-flight mass spectrometry (UHPLC-HR-TOFMS) method was used for the analysis of the samples. The chromatographic and mass spectrometric conditions were similar to those described previously^21^. The monitored compound signals were extracted at *m/z*-window of 5 mDa. The median mass accuracy and isotopic pattern match of the measured compounds were 0.3 mDa and 4, respectively.

### Statistics

Data obtained from the cytotoxicity and mitochondrial respiration experiments were analyzed using one-way ANOVAs with Fisher’s Least Significant Difference *post hoc* tests. Data of the cytotoxicity experiments based on the LDH release observed in control wells (untreated cells) were normalized. Pairwise comparisons were made using the independent samples by the paired t test to analyze data from the 4-MMC ± GBR12909 LDH release experiments and also to analyze those data from the TNFα and HPLC DA experiments. Statistical significance was set at values *p* < 0.05. All data are presented as mean ± SEM, unless otherwise specified.

## Results

### Validation of dopamine neuron-like phenotype

The DA-ergic phenotype were validated if the levels of cellular DA were measured in both undifferentiated and RA-TPA differentiated cells. DA was detected in RA-TPA differentiated cells, but not in the undifferentiated cells. Further, a 3-day treatment with the TH inhibitor α-methyl-para-tyrosine (0.5 mM) reduced DA-levels to below quantifiable limits (Supplementary Fig. 1). This increase in DA levels was associated with a significant increase in the expression of TH, and certain other DA-related genes (Supplementary Table 1).

### Cytotoxicity and mitochondrial respiration following cathinone- and amphetamine-treatments

#### Cytotoxicity

**Figure 1:**
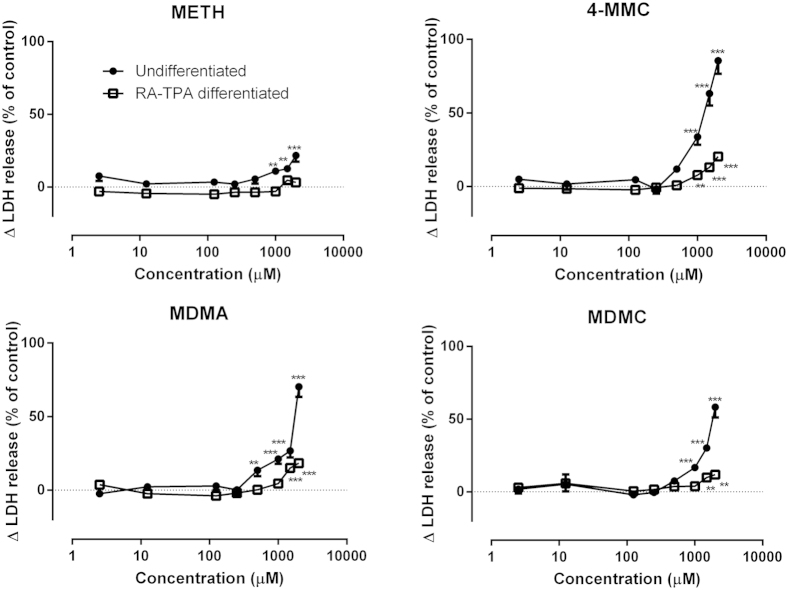
The amounts of lactate dehydrogenase (LDH) released into the medium from SH-SY5Y cells during a 48 h treatment as a percentage of the control values for METH, 4-MMC, MDMA or MDMC (2.5–2000 μM) in undifferentiated (black circles) and RA-TPA differentiated (squares). The graphs show the percentage change in total LDH released into the medium as a function of increasing drug concentrations, compared to untreated cells. Only significant differences larger than 5% compared to control are marked *p < 0.05; **p < 0.01; ***p < 0.001. N = 5–8 per group.

The effects of a 48 h treatment with varying doses (2.5–2000 μM) of METH, 4-MMC, MDMA or MDMC on the amounts LDH released into the growth media for both undifferentiated and RA-TPA differentiated cells is shown in Fig. 1. For undifferentiated cells, a one-way ANOVAs revealed that METH (F_8 55_ = 5.89, *p* < 0.001), 4-MMC (F_8 47_ = 42.80, *p* < 0.001), MDMA (F_8 55_ = 41.90, *p* < 0.001) and MDMC (F_8 47_ = 36.34, *p* < 0.001) all significantly affected LDH release. *Post hoc* testing showed that all drugs dose-dependently increased LDH release. For RA-TPA differentiated cells, one-way ANOVAs also revealed significant effects on LDH release following a 48 h treatment with varying doses (2.5–2000 μM) of METH (F_8 55_ = 3.79, *p* < 0.01), 4-MMC (F_8 47_ = 14.57, *p* < 0.001), MDMA (F_8 55_ = 18.60, *p* < 0.001) and MDMC (F_8 47_ = 3.00, *p* < 0.05), with *post hoc* tests showing dose-dependent increases in cytotoxicity with higher drug concentrations for all drugs besides METH.

#### Mitochondrial respiration

**Figure 2:**
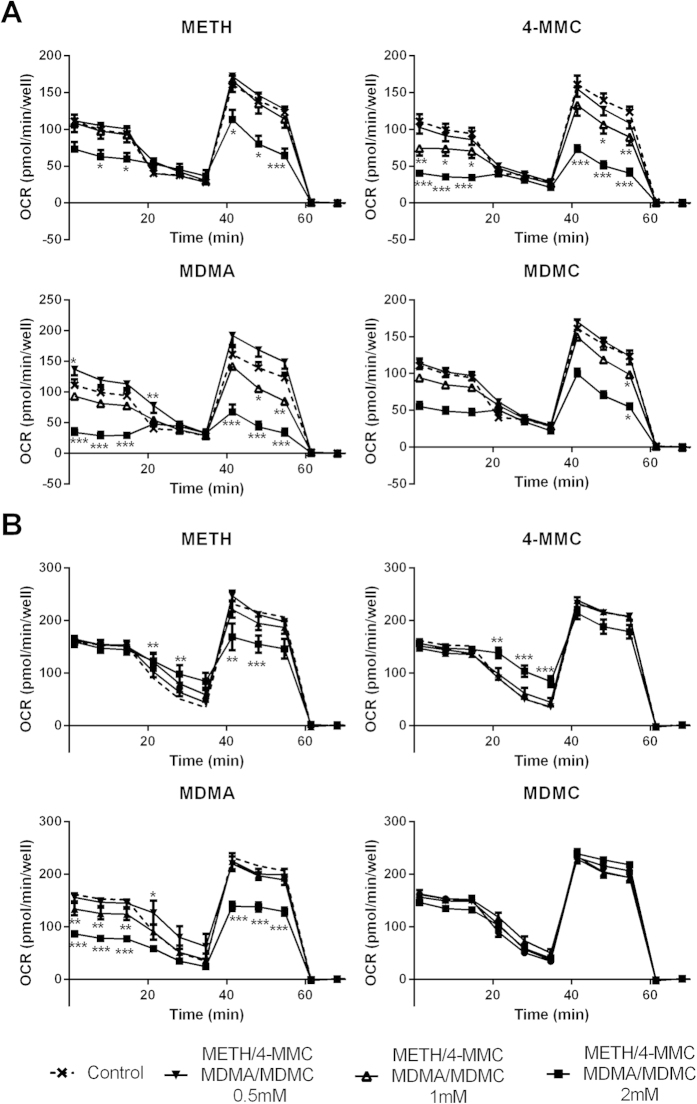
Oxygen consumption rate (OCR) of undifferentiated cells (A) or RA-TPA differentiated cells (B) that had been previously exposed to one of the following METH, 4-MMC, MDMA or MDMC (0.5–2.0 mM) for 24h. The graphs show the baseline OCR (prior to any injections) the OCR after injection of the complex IV inhibitor oligomycin (Oli), the uncoupler carbonyl cyanide 4-(trifluoromethoxy)phenylhydrazone (FCCP) and a combined injection of rotenone + antimycin A (Rot + Ama) (final concentration of all compounds was 1 μM). Measurements were performed using the Seahorse XFe96 instrument. *p < 0.05; **p < 0.01; ***p < 0.001 compared to control (shown as a dashed line). N = 5–8 per group.

The effects of a 24 h treatment with varying doses (0.5–2 mM) of METH, 4-MMC, MDMA or MDMC on mitochondrial respiration in intact cells were determined. Differences in baseline respiration and respiration following injection of oligomycin, FCCP and rotenone + antimycin A into the assay medium were assessed using separate one-way ANOVAs with *post hoc* tests and the resulting respiratory graph plots are shown in Fig. 2A (undifferentiated cells) or Supplementary Fig. 2B (RA-TPA differentiated cells). These graphs show that various drug treatments caused perturbations in mitochondrial respiration, although the effects are more pronounced in the undifferentiated cells.

### Mechanisms involved in cytotoxicity

Both amphetamines and cathinones exert a clear dose-dependent effect on cytotoxicity and mitochondrial respiration, we therefore investigated the involvement of several factors known to play a role in amphetamine toxicity.

#### Dopamine and DAT

**Figure 3:**
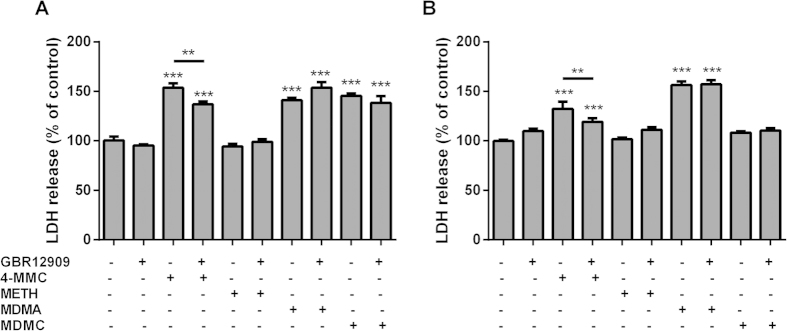
The effect of GBR12909 on LDH release from SH-SY5Y cells in response to 4-MMC, METH, MDMC or MDMA (all 2 mM) in (A) undifferentiated cells and (B) RA-TPA differentiated cells. Cells were treated with GBR12909 or the vehicle for 1 h prior to exposure to 4-MMC, METH, MDMC or MDMA. **p < 0.01; ***p < 0.001. N = 6–10 per group.

The effect of DAT blockade on LDH release following a 48 h treatment with METH, 4-MMC, MDMA and MDMC (all 2 mM) was investigated using the DAT blocker GBR12909 (Fig. 3A). A one-way ANOVA showed a significant effect of treatment (F_9 65_ = 39.08, *p* < 0.001) on LDH release. *Post hoc* testing indicated that 4-MMC, MDMA and MDMC all significantly increased LDH release compared to untreated cells. However, only the LDH release induced by 4-MMC was partially but significantly inhibited by co-treatment with GBR12909 (t_8_ = 3.23, *p* < 0.01). Similarly, a significant effect of DAT-blockade was found in RA-TPA differentiated cells (F_9 64_ = 43.12, *p* < 0.001) with *post hoc* testing showing significant reduction of 4-MMC toxicity due to DAT-blockade with GBR12909 (Fig. 3B). Conversely, co-treatment with the TH inhibitor α-methyl-para-tyrosine did not significantly affect the toxicity of 4-MMC or METH in RA-TPA differentiated cells (F_5 35_ = 21.15, *p* < 0.001). *Post hoc* testing showed no difference in cytotoxicity between cells treated with or without α-methyl-para-tyrosine (Supplementary Fig. 3).

#### ROS and oxidative stress

**Figure 4:**
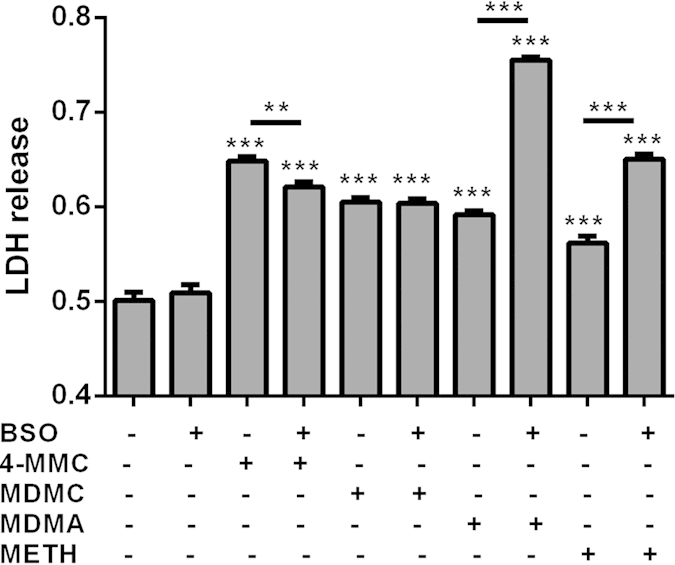
The effect of buthionine sulfiximine (BSO 4 mM) on LDH release by 4-MMC, MDMC, MDMA or METH (all 2 mM). Cells were treated with BSO for 24 h prior to drug exposure as well as during drug the 48 h drug exposure. **p < 0.01; ***p < 0.001. N = 7–8 per group.

The production of ROS were determined by measuring levels of H_2_O_2_ produced by solutions of one of the following: 20 mM METH, 4-MMC, MDMA and MDMC and is shown in Supplementary Fig. 4. A steady increase in fluorescence over time can be observed after the addition of the cathinones 4-MMC and MDMC, but not after the addition of amphetamines METH, MDMA or the vehicle (H_2_O) control. In the absence of HRP, none of the drugs produced an increase in fluorescence, which indicated that the effect was not due to a direct reaction between the cathinones and Amplex Red. We subsequently investigated the involvement of oxidative stress-mediated processes in the cytotoxic response of SH-SY5Y cells to amphetamines and cathinones by depleting cellular glutathione with 4 mM BSO (Fig. 4.) A significant effect of treatment was found on LDH release (F_9 78_ = 158.6, p < 0.001). Post hoc testing revealed that BSO strongly increased the LDH release due to METH and MDMA, while in fact reducing 4-MMC-induced LDH release and having no effect on MDMC-induced LDH release.

#### Bcl-2 involvement

**Figure 5:**
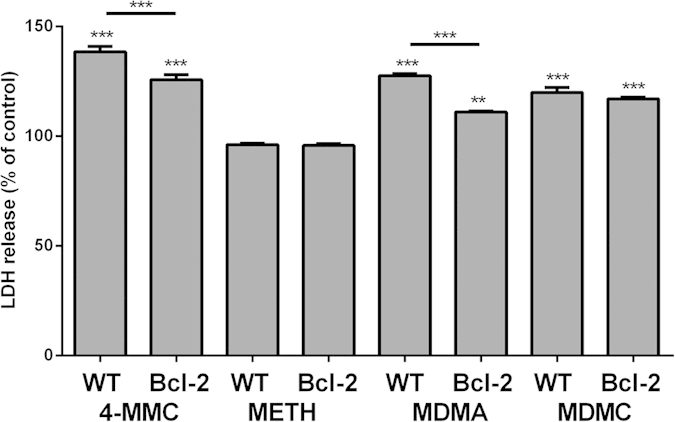
Effect of Bcl-2 overexpression on cytotoxicity (LDH release into medium) induced by a 48h treatment of SH-SY5Y with 4-MMC, METH, MDMC or MDMA (all 2 mM). Data are expressed as a percentage LDH release compared to respective (normal or Bcl-2 overexpressing) untreated control cells. **p < 0.01; ***p < 0.001. N = 6 per group.

The involvement of the anti-apoptotic mitochondrial Bcl-2pathway in drug-induced cytotoxicity was investigated by comparing the amount of LDH release in normal and Bcl-2 overexpressing SH-SY5Y cells following a 48 h treatment with 2 mM METH, 4-MMC, MDMA or MDMC (Fig. 5). The percentage LDH release compared to untreated cells was calculated for both normal and Bcl-2 overexpressing cells. A one-way ANOVA of the resulting data demonstrated a significant effect (F_8 53_ = 84.45, *p* < 0,001), and subsequent *post hoc* tests showed that Bcl-2/BCL-2 overexpression partially but significantly attenuated both 4-MMC and MDMA cytotoxicity, but did not affect MDMC-induced cytotoxicity.

#### TNFα

**Figure 6:**
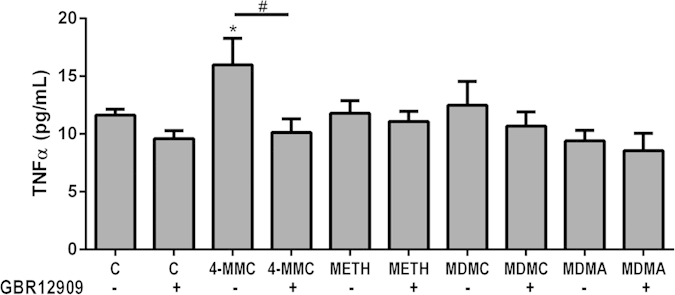
The effect of a 48 h treatment with 2 mM 4-MMC, METH, MDMC or MDMA alone or in combination with 1 μM GBR12909 on levels of tumor necrosis factor α (TNF α) in cellular growth medium. *p < 0.05, Co-treatment with GBR12909 tended to attenuate the 4-MMC-induced increase in TNFα levels #p = 0.064. N = 4–10 per group.

The effects of individual treatments 2 mM 4-MMC, METH, MDMC or MDMA alone or in combination with GBR12909 on medium TNFα levels are shown in Fig. 6. Treatment significantly affected TNFα levels (F_9 45_ = 2.66, p < 0.05), and *post hoc* testing revealed that only 4-MMC treatment significantly increased medium TNFα levels. Co-treatment with GBR12909 tended to attenuate the 4-MMC-induced increase in TNFα levels (*p* = 0.064).

#### p53

**Figure 7:**
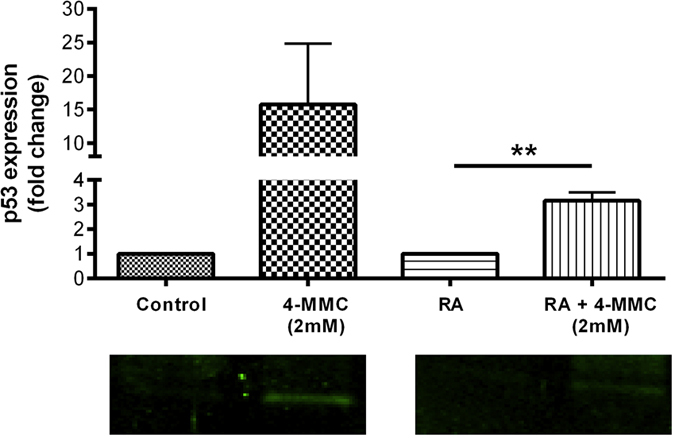
Expression of p53 in control and RA-treated SH-SY5Y cells with or without 4-MMC (2 mM) after 48 hours. Relative fluorescence intensity values were obtained from western blots of samples from three independent experiments. Data is presented as the mean fold change + SEM (N = 3) when comparing 4-MMC-treated samples with representative controls receiving vehicle only (control) or RA + vehicle (RA). **p < 0.01.

The effects of a 48 h treatment with 4-MMC on p53 protein levels is shown in Fig. 7. A significant induction of p53 was observed in cells treated with 4-MMC and RA, compared to cells treated only with RA (t_4_ = 6.47, *p* < 0.01). A large variance was observed in p53 levels of control cells treated with 4-MMC and the observed increase in p53 levels was therefore not significant (t_4_ = 1.62, ns).

### Detection of methylbenzamide breakdown products

We measured DMBA and MDMBA levels 0 and 48 h after dissolving 2 mM 4-MMC or MDMC in pH 12 phosphate-buffered saline in order to demonstrate the spontaneous breakdown of cathinones over time. The quantity of DMBA was also measured in the SH-SY5Y medium following 4-MMC treatment to demonstrate that breakdown also occurs under physiological conditions.

#### pH 12 phosphate buffer

We performed UHPLC-HR-TOFMS on highly alkaline samples of 4-MMC and MDMC in order to compare the findings with those of Tsujikawa *et al.*^13^. The UHPLC-HR-TOFMS data indicated an accumulation of both DMBA and MDMBA breakdown products at 48 hours, which is in accordance with the findings reported by Tsujikawa *et al.*^13^ under alkaline pH conditions. The ion chromatograms are shown in Supplementary Fig. 5.

#### SH-SY5Y growth medium

**Figure 8:**
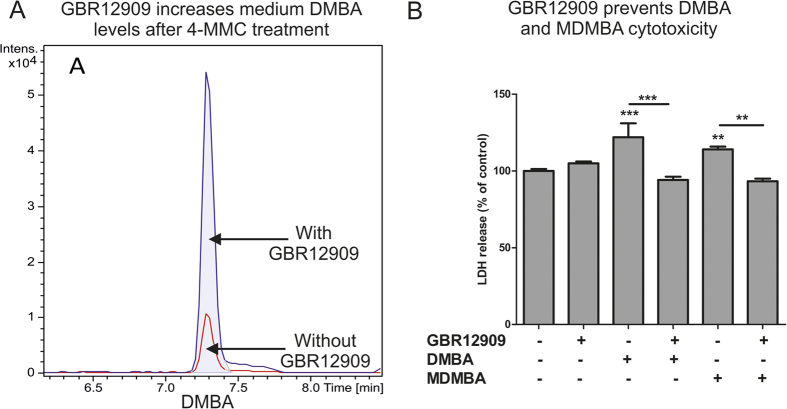
(**A**) Extracted ion chromatograms of DMBA in SH-SY5Y cell growth medium following an incubation of 48 h for SH-SY5Y cells with 4-MMC in the presence (blue) or absence(red) of 1 μM GBR12909. (**B**) The effect of a 48 h treatment with DMBA or MDMBA either alone, or in combination with the DAT blocker GBR12909, which fully blocked the cytotoxicity produced by both drugs. GBR12909 was applied 30 m before addition of DMBA or MDMBA. **p < 0.01; ***p < 0.001. N = 3–10 per group.

The amount of DMBA breakdown product in SH-SY5Y growth medium following a 48 h treatment of SH-SY5Y cells with 4-MMC in the presence or absence of GBR12909 is presented in Fig. 8A and shows a clear increase in medium DMBA levels when GBR12909 is present. These data suggest that DMBA may also be produced under physiological conditions and that DAT inhibition (as evidenced here by the effect of GBR12909) may prevent its cellular uptake.

### Cytotoxicity and mitochondrial respiration following methylbenzamide-treatment

#### Cytotoxicity

The amount of LDH released into the medium following a 48 h treatment with 2 mM DMBA or MDMBA, alone or in combination with GBR12909 was measured and is shown in Fig. 8B. A one-way ANOVA revealed a significant effect of treatment on LDH release (F_5 25_ = 11.52, *p* < 0.001). *Post hoc* tests revealed that both DMBA and MDMBA caused a significant increase in LDH release compared to the untreated cells and that this increase was fully blocked by co-treatment with DAT blocker GBR12909.

#### Mitochondrial respiration

**Figure 9:**
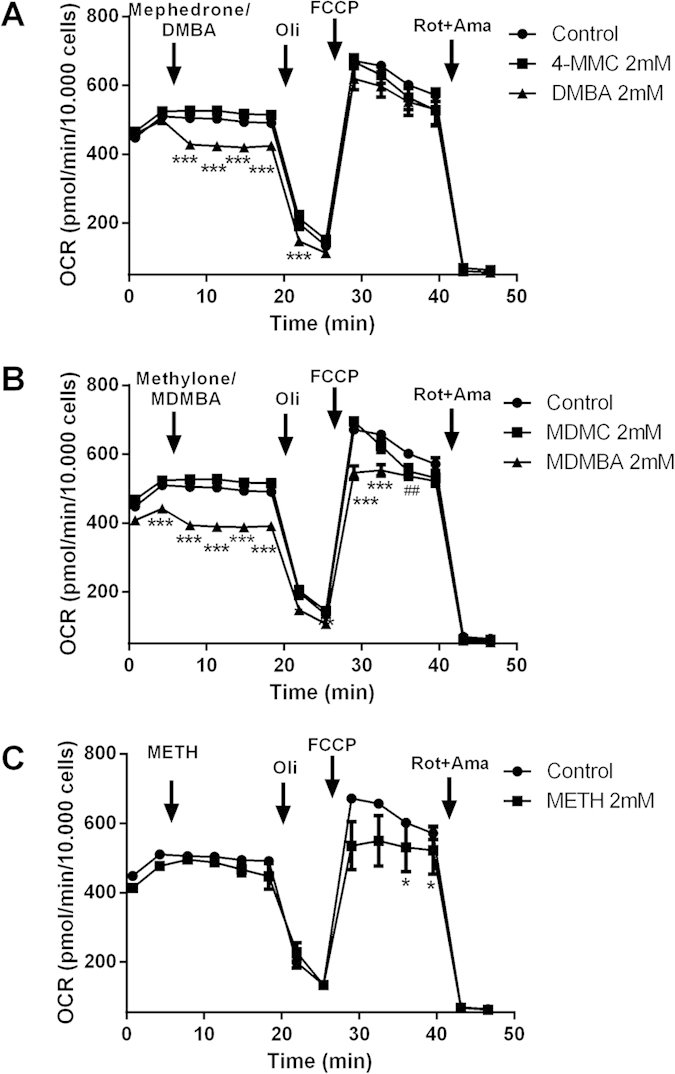
Baseline oxygen consumption rate (OCR) and OCR following treatments with (A) 4-MMC or DMBA; (B) with MDMC or MDMBA or (C) with METH (all 2 mM), all followed by treatments with the complex V inhibitor oligomycin (Oli, final concentration 2 μM), the uncoupler carbonyl cyanide 4-(trifluoromethoxy)phenylhydrazone (FCCP, final concentration 10 μM) and a combined treatment of the mitochondrial inhibitors rotenone and antimycin-A (Rot + Ama, final concentration 2 μM each). The assay was performed using the Seahorse Bioscience XFe96 analyzer in cells that had been permeabilized by recombinant perfringolysin O (2 nM) directly prior to the start of the assay. *p < 0.05; **p < 0.01; ***p < 0.001 compared to control. N = 9–12 per group.

The effects of 4-MMC and DMBA; MDMC and MDMBA in addition to METH on mitochondrial respiration were subsequently assessed in permeabilized SH-SY5Y cells. The respiratory rate plots (Fig. 9) show differences in baseline OCR before and during the 10 min incubation with the drugs. OCR following injection of oligomycin, FCCP and rotenone + antimycin A into the assay medium are also shown in Fig. 8. These data show that DMBA and MDMBA cause a rapid reduction in both baseline and maximum respiration. METH does not affect baseline respiration, but eventually produces a reduction in the maximum respiration at two time points. Conversely, the cathinones 4-MMC and MDMC have little effect on respiration, except from a slight reduction in maximum respiration after MDMC, but only at one time point.

## Discussion

We showed that both amphetamines and cathinones can produce a dose-dependent cytotoxic effect and decrease mitochondrial respiration. The cytotoxic effect of cathinone 4-MMC is larger than that of METH, and can be partially prevented by DAT blockade and overexpression of mitochondria-protective anti-apoptotic Bcl-2. Meanwhile, 4-MMC also caused induction of p53. However, depletion of DA *per se* did not significantly affect toxicity. We found that cathinones, spontaneously produce ROS in aqueous solution as measured by the Amplex Red/ROS accumulation assay, whereas amphetamines do not. Furthermore, depletion of cellular glutathione strongly increases amphetamine toxicity, but has no effect or actually decreases, cathinone toxicity. We also found that the conversion of 4-MMC to its corresponding methylbenzamide breakdown product can occur under physiological conditions, and that treatment with methylbenzamides alone is sufficient to produce cytotoxicity and decrease mitochondrial respiration.

**Figure 10:**
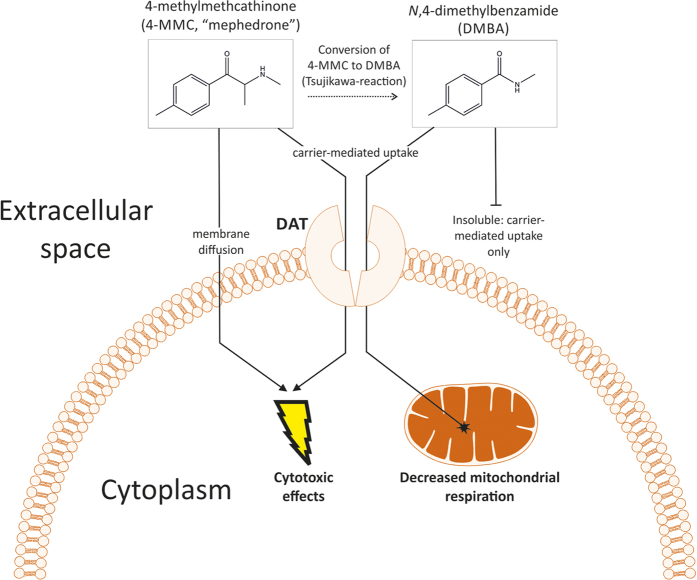
Proposed model for the observed effects of 4-MMC and DMBA in cells. In aqueous solution, the cathinone 4-MMC is gradually broken down into the methylbenzamide DMBA, which is a step we have dubbed the Tsujikawa-reaction after the researcher who first described this (Tsujikawa *et al.* 2012). Both 4-MMC and DMBA are taken up into the cytoplasm via the DAT. Next to the carrier-mediated uptake, 4-MMC can gain entry to the cell simply via diffusion through cellular membranes. Hence, DAT inhibition does not fully prevent the cytotoxic effects of 4-MMC. DMBA, however, cannot diffuse across membranes and is fully dependent on the DAT carrier mechanism for its cellular uptake. Once inside the cell, DMBA induces a rapid decrease in mitochondrial respiration and increases cytotoxicity in the cytoplasm.

The breakdown of cathinones into their corresponding methylbenzamides has been described previously to occur under conditions of high pH, or in the presence of electron acceptors^11^,^13^. Here, we showed that the breakdown can also occur under normal physiological conditions in cellular growth media with cells present. This observation is of particular interest because methylbenzamides themselves appear to cause cytotoxicity and affect mitochondrial respiration. Detailed quantitative measurements of DMBA and MDMBA and their formation under physiological conditions both *in vitro* and *in vivo* are therefore warranted. The cytotoxicity observed after cathinone treatment can be separated into multiple components: one that is directly due to the effect of the cathinones themselves and a second component that is due to their methylbenzamide breakdown products (represented schematically in Fig. 10). Furthermore, interactions between the two may also occur. Although it is difficult to quantify the exact contribution of each metabolite makes to the total toxicity using the experimental design described here, our present study’s findings do support the conclusion that cathinones themselves may be less toxic than suggested by continuous-treatment exposure *in vitro*. This conclusion is supported by *in vivo* data on neurotoxicity^3^,^22^. An important two-part question therefore is to what extent does the observed *in vitro* breakdown of cathinones into their respective methylbenzamides products proceed, and can the toxicity of these methylbenzamides products be reproduced in *in vivo* studies? The breakdown appears to be a relatively slow continuous process as opposed to a rapid reaction, thus it is likely that the effects of the breakdown products *in vivo* may be limited to periods of very long exposure to cathinones, such as multi-day binge use, and not be evident after only one or a few doses/treatments with the drug. Furthermore, the occurrence of these breakdown products may be helpful in explaining why cathinone toxicity can be precipitated by certain factors, including hyperthermia, as these factors may influence the rate at which cathinones are converted to methylbenzamides^9^,^23^.

The observed increase in TH, mrNA and DA levels confirm the successful induction of a DA neuron-like phenotype using the RA-TPA differentiation protocol described by Presgraves and colleagues^16^. It was previously shown that the differentiation by RA-TPA treatment also produces a more resistant cellular phenotype, which has been linked to the upregulation of Nrf2 and NAD(P)H quinone oxidoreductase 1, both of which play an important role in the endogenous antioxidant response and redox balance^24^. Our results are largely consistent with this as both the cytotoxic response and the decrease in mitochondrial respiration we observed after drug treatments was generally lower in RA-TPA differentiated cells, compared to undifferentiated cells. The effects of METH, 4-MMC and MDMC on cytotoxicity and of 4-MMC and METH on basal and maximum respiration in undifferentiated cells correspond closely to those reported by us previously^11^. The enhanced sensitivity of undifferentiated cells limited us to performing subsequent experiments that investigated the involvement of Bcl-2, ROS and TNFα in cytotoxicity in undifferentiated cells in addition to the effect of DA-like phenotype in RA-TPA differentiated cells.

It has been previously shown that the overexpression of Bcl-2 protein reduces METH toxicity^25^ and that the *BCL-2* family of genes may also play a role in regulating METH toxicity *in vivo*, in particular when very high drug doses are administered^26^,^27^. We showed that the protective effects of the overexpression of Bcl-2 found in this study extend to the treatment with high doses of cathinones *in vitro* as well. Conversely, we observed that the cathinones 4-MMC and MDMC, resulted in a spontaneous production of ROS when in an aqueous solution, whereas this did not occur for the amphetamines METH and MDMA. It is therefore interesting that we observed that glutathione depletion with BSO had no effect on cathinone cytotoxicity as it did on cytotoxicity produced by METH and MDMA. This points towards an interesting difference in the interaction between amphetamines and cathinones on oxidative stress-related toxicity that warrants further investigation. The involvement of TNFα and p53 in amphetamine toxicity has previously been demonstrated both *in vitro* and *in vivo*^28^,^29^,^30^,^31^,^32^, and in the present study we demonstrated that these factors may also be involved in cathinone toxicity.

One limitation of this study is that the drug concentrations of up to 2 mM that were used to induce cytotoxicity and mitochondrial respiratory dysfunction, are much higher than those observed in human amphetamine users. However, this concentration is similar to concentrations used in previous studies on amphetamine toxicity^33^,^34^,^35^,^36^. A second limitation is that although SH-SY5Y cells are commonly used as *in vitro* models for studying DA-related neurological phenomena such as Parkinson’s disease and amphetamine neurotoxicity^37^, they are neuroblastoma cells. For these reasons, the results may not be directly translatable to humans but should be viewed mainly as a model for understanding mechanisms that may potentially play a role in the response of the human nervous system to amphetamines exposure.

Our results provide an important first insight into the mechanisms of cathinone cytotoxicity. These data emanate from *in vitro* experiments and they pave the way for further investigations into cathinone metabolism *in vivo* in addition to a broader evaluation of the specific contribution their metabolic breakdown products make to the total cytotoxicity. Therefore, future work is warranted that assesses the contribution of methylbenzamides and their potential interactions with cathinones in producing neurotoxicity under various circumstances *in vivo*.

## Conclusion

Cathinones slowly but spontaneously degrade into their respective methylbenzamide breakdown products in aqueous solutions. The methylbenzamide products themselves possess toxic properties and contribute to the overall observed cathinone toxicity. When discriminating between the toxic effects of cathinones and their methylbenzamide breakdown products, the specific contribution of the cathinones *per se* may be quite limited. The breakdown of cathinones to methylbenzamides as well as the differential effects of cathinones and amphetamines on oxidative stress-related toxicity may contribute to an understanding of the toxicological differences between cathinones and amphetamines under various conditions *in vivo*, in addition to any discrepancies observed between *in vivo* and *in vitro* data.

## Additional Information

**How to cite this article**: den Hollander, B. *et al.* Mitochondrial respiratory dysfunction due to the conversion of substituted cathinones to methylbenzamides in SH-SY5Y cells. *Sci. Rep.* **5**, 14924; doi: 10.1038/srep14924 (2015).

## Supplementary Material

Supplementary Information
