# Supplementary material for: Mitochondrial respiratory dysfunction due to the conversion of substituted cathinones to methylbenzamides in SH-SY5Y cells
Source: Sci Rep. 2015 Oct 14;5:14924. doi: 10.1038/srep14924 (PMC4604489; doi:10.1038/srep14924)
Supplement: Supplementary Information [file srep14924-s1.pdf]

Mitochondrial respiratory dysfunction due to the conversion of substituted cathinones to methylbenzamides in SH-SY5Y cells

- Supplementary Information -

Bjørnar den Hollander*; Mira Sundström† (mira.sundstrom@helsinki.fi); Anna Pelander† (anna.pelander@helsinki.fi); Antti Siltanen* (antti.siltanen@helsinki.fi); Ilkka Ojanperä† (ilkka.ojanpera@helsinki.fi); Eero Mervaala*MD (eero.mervaala@helsinki.fi); Esa R. Korpi* (esa.korpi@helsinki.fi); Esko Kankuri* (esko.kankuri@helsinki.fi).

* Department of Pharmacology, Faculty of Medicine, Biomedicum Helsinki, Haartmaninkatu 8, FI-00014 University of Helsinki, Finland. † Department of Forensic Medicine, Kytösuontie 11, FI-00014 University of Helsinki, Finland.

Supplementary Figure 1

Effect of differentiation protocol on dopamine levels

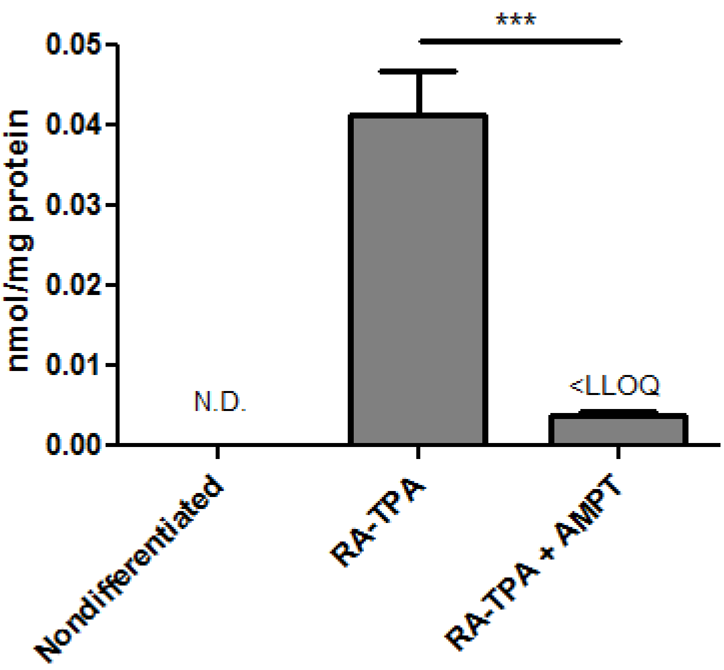

Fig. S1 Levels of DA measured in undifferentiated cells, RA-TPA differentiated cells and RA-TPA differentiated cells 0.5 mM of the tyrosine hydroxylase inhibitor α-methyl-para-tyrosine (AMPT) present in the medium during the last 3 days of differentiation. ND not detected; <LLOQ below the lower limit of quantification. N = 4 per group.

Supplementary Table 1

Effect of differentiation on the expression of dopamine-related genes

**Gene expression in undifferentiated and RA-TPA-differentiated cells**	
Upregulated in RA-TPA	
Name	Fold change
Tyrosine hydroxylase	132.9
D1 receptor	8.0
D2 receptor	2.0
D4 receptor	1.5
Downregulated in RA-TPA	
Name	Fold change
DAT	3.5
VMAT-2	3.0
MAO-B	2.7

Table S1 Expression of selected DA-related genes in undifferentiated cells and RA-TPA differentiated cells.

Supplementary Figure 2

Mitochondrial respiration in RA-TPA differentiated cells

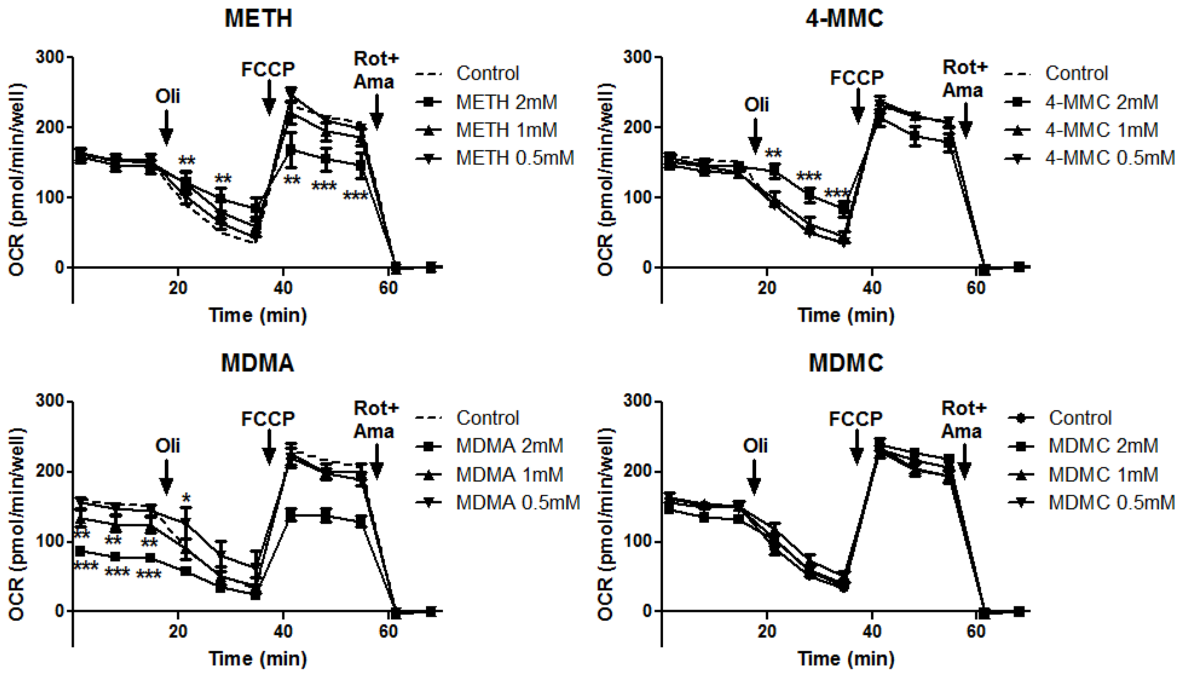

Fig. S2 Oxygen consumption rate (OCR) of RA-TPA differentiated SH-SY5Y cells following a 24 h exposure to either METH, 4-MMC, MDMA or MDMC (0.5 - 2.0mM). The graphs show the baseline OCR (prior to any injections) well as the OCR after injection of the complex IV inhibitor oligomycin (Oli), the uncoupler carbonyl cyanide 4-(trifluoromethoxy)phenylhydrazone (FCCP) and finally a combined injection of rotenone + antimycin A (Rot+Ama) (final concentration of all compounds: 1µM). Measurements were performed using the Seahorse XFe96 instrument. * $p < 0.05$; ** $p < 0.01$; *** $p < 0.001$ compared to control (shown as a dashed line). N = 5 - 8 per group.

Supplementary Figure 3

Effect of α-methyl-para-tyrosine on cytotoxicity

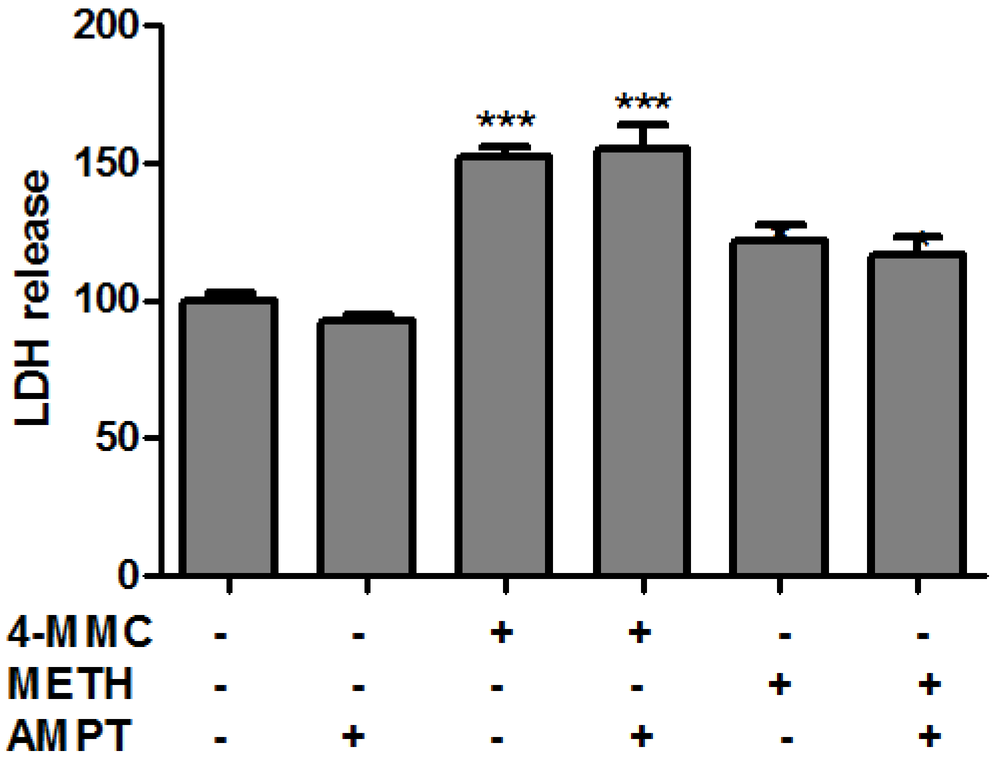

Fig. S3 The effect of the TH inhibitor α-methyl-para-tyrosine on LDH release induced by a 48 h treatment with 2 mM 4-MMC or METH in RA-TPA differentiated cells. Cells were treated with α-methyl-para-tyrosine during the last 3 days of differentiation as well as during the 48 h drug treatment. N = 6 per group.

Supplementary Figure 4

Spontaneous ROS production from cathinones

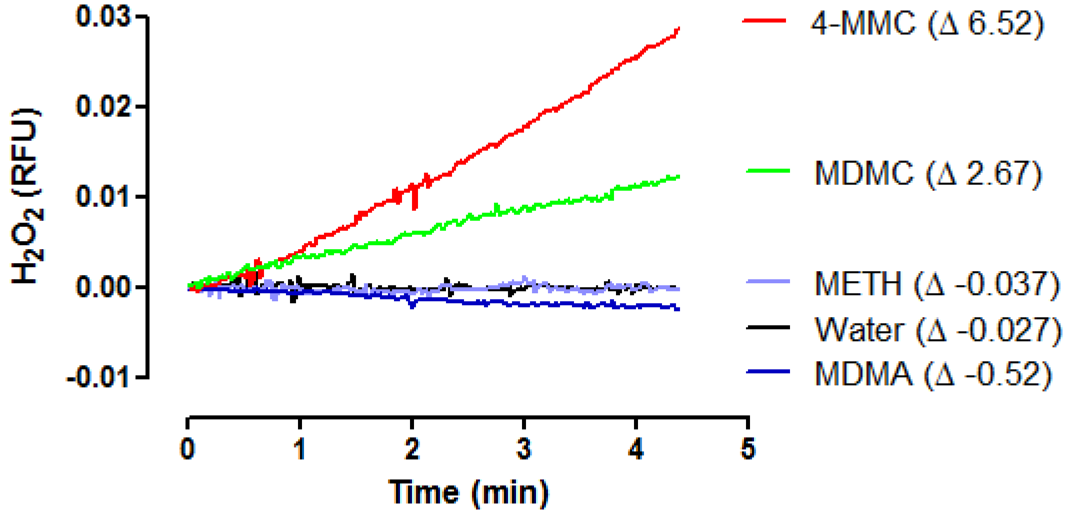

Fig. S4 Cumulative H_2O_2 production (relative fluorescence units, RFU) after the addition of water or METH, MDMA, 4-MMC and MDMC (all 20mM) were determined using an Amplex Red - Horseradish peroxidase assay in the Oxygraph-2k instrument fitted with a fluorescence module. Change in H_2O_2 production (Dslope after/before-injection) is indicated in paranthesis.

Supplementary Figure 5

Production of methylbenzamide breakdown products in alkaline pH

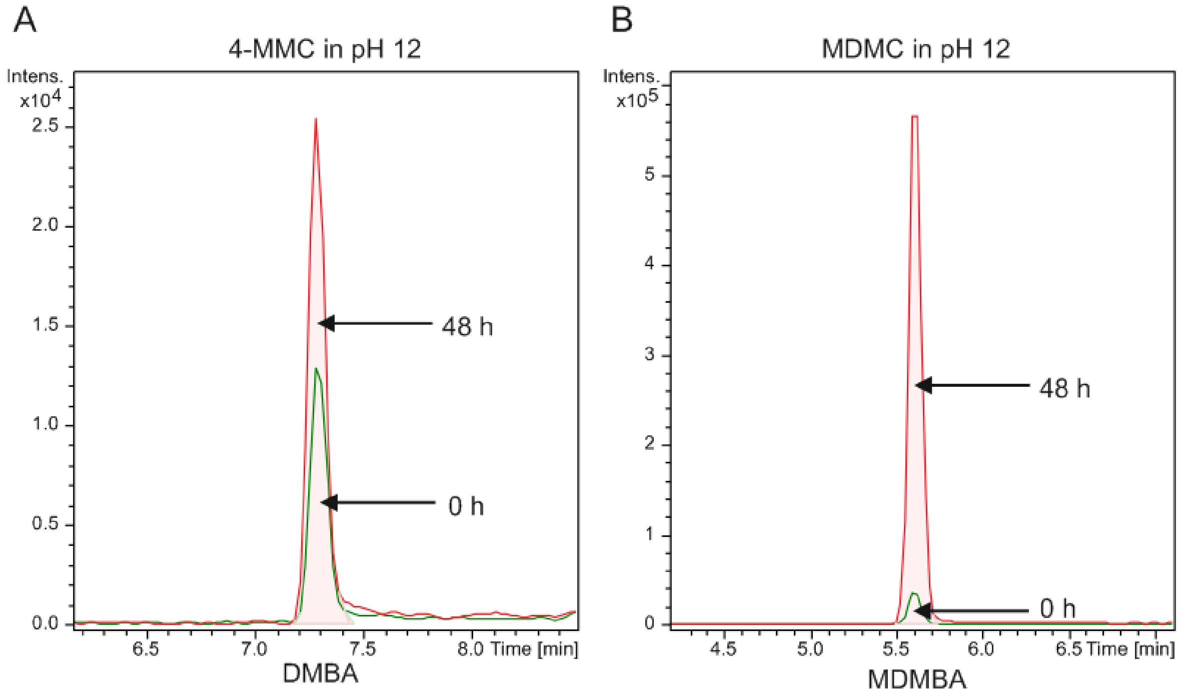

Fig. S5 Extracted ion chromatograms of DMBA (A) and MDMBA (B) in pH 12 after incubating 4-MMC (A) or MDMC (B) for 0 h (green) and 48 h (red). As the incubation time increases the peak intensity of these breakdown products also increases.
